# A Metabolomics Study of the Effects of Eleutheroside B on Glucose and Lipid Metabolism in a Zebrafish Diabetes Model

**DOI:** 10.3390/molecules29071545

**Published:** 2024-03-29

**Authors:** Xuelian Dong, Qiang Chen, Wenyan Chi, Zhidong Qiu, Ye Qiu

**Affiliations:** Institute of College of Pharmacy, Changchun University of Chinese Medicine, Changchun 130117, China; dongxuelian1979@126.com (X.D.); chenqiang990105@163.com (Q.C.); xinyu.325@foxmail.com (W.C.); qzdcczy@163.com (Z.Q.)

**Keywords:** eleutheroside B, diabetes mellitus, zebrafish, metabolomics

## Abstract

(1) Background: Diabetes is a common metabolic disease that seriously endangers human health. In the present study, we investigated the therapeutic effects of the active ingredient Eleutheroside B (EB) from the traditional Chinese medicine Eleutheroside on diabetes mellitus in a zebrafish model. Concomitant hepatic injury was also analysed, along with the study of possible molecular mechanisms using metabolomics technology. This work should provide some theoretical references for future experimental studies. (2) Methods: A zebrafish diabetes model was constructed by soaking in a 1.75% glucose solution and feeding a high-fat diet. The intervention drug groups were metformin (100 μg∙mL^−1^) and EB (50, 100, and 150 μg∙mL^−1^) via water-soluble exposure for 30 days. Glucose, TG, TC, LDL-C, and HDL-C were evaluated in different treatment groups. GLUT4 protein expression was also evaluated in each group, and liver injury was observed by HE staining. Metabolomics techniques were used to investigate the mechanism by which EB regulates endogenous markers and metabolic pathways during the development of diabetes. (3) Results: All EB treatment groups in diabetic zebrafish showed significantly reduced body mass index (BMI) and improved blood glucose and lipid profiles. EB was found to upregulate GLUT4 protein expression and ameliorate the liver injury caused by diabetes. Metabolomics studies showed that EB causes changes in the metabolic profile of diabetic zebrafish. These were related to the regulation of purine metabolism, cytochrome P450, caffeine metabolism, arginine and proline metabolism, the mTOR signalling pathway, insulin resistance, and glycerophospholipid metabolism. (4) Conclusions: EB has a hypoglycaemic effect in diabetic zebrafish as well as significantly improving disorders of glycolipid metabolism. The mechanism of action of EB may involve regulation of the mTOR signalling pathway, purine metabolism, caffeine metabolism, and glycerophospholipid metabolism.

## 1. Introduction

Diabetes mellitus (DM) is a chronic metabolic disease characterised mainly by elevated hyperglycaemia. DM poses a serious threat to human health, and its incidence rate continues to rise worldwide. The disease is characterised by multiple complications, high morbidity, and difficulty in eradicating. DM is the third most common non-communicable disease, after cancer and cardiovascular disease [1]. In 2019, about 463 million people worldwide suffered from DM, with this number expected to increase to 578 million by 2030 [2]. The common forms of diabetes are referred to as type 1 and type 2. Type 1 DM is an autoimmune disease with absolute insulin deficiency due to damaged pancreatic β-cells. It usually occurs in children and early adolescents, and accounts for about 5% to 10% of all diabetes cases. Type 2 DM (T2DM) is a chronic metabolic disease characterised by insulin resistance (IR) and elevated blood glucose levels. It accounts for >90% of all diabetic patients and is common in the elderly. However, in recent years, the proportion of young people and children with T2DM has been increasing [3]. T2DM is thought to result from the interaction between genetic and environmental factors (e.g., diet, lifestyle, and environmental pollution) [4,5]. T2DM is characterised by hyperglycaemia, IR, and impaired insulin secretion, leading to disorders of glucose–lipid metabolism and complications that greatly reduce the patients’ quality of life and even endanger their lives in severe cases [6]. IR refers to the reduced sensitivity and responsiveness of insulin-target organs or target tissues to insulin and the decreased efficiency of glucose uptake and utilisation. This results in abnormalities of glucose–lipid metabolism, leading to normal amounts of insulin that produce biological effects below physiological levels [7].

The liver is an important metabolic organ in the human body and is involved in the regulation of blood glucose and the storage and distribution of sugar. It is an important target organ for the chronic complications of DM. In a state of high glucose, there is a disturbance in glucose metabolism, a reduction in glucose tolerance, and an induction of IR. The strong compensatory function of the liver makes it difficult to detect DM combined with liver lesions in the early stages, and the damaged liver further exacerbates disorders of glucose metabolism [8]. IR leads to a reduction in the metabolic function of liver cells, resulting in the accumulation of excessive lipids in the liver. This causes oxidative stress and inflammatory responses in hepatocytes, which ultimately aggravate liver damage [9]. Oxidative stress is a significant mechanism in the development of diabetic complications, and the liver is the organ most vulnerable to it [10]. Reactive oxygen species (ROS), a marker of oxidative stress, cause biomolecule oxidation, DNA damage, reversible or irreversible changes in proteins, and lipid peroxidation products, which further harm liver tissue [11]. IR, metabolic syndrome, obesity, adipose tissue dysfunction, and the secretion of inflammatory substances cause the body to be in a state of chronic inflammation for a long period of time. The metabolic functions of the liver will therefore continue to be harmed due to prolonged exposure to a chronic inflammatory environment [12].

A major regulator of cellular metabolism is mTOR, which is a serine/threonine kinase. The mTOR pathway is regulated by a variety of cellular signals, including mitotic growth factor, hormones such as insulin, glucose, and amino acids, cellular energy levels, and stress conditions. mTOR has the ability to promote metabolism, participate in apoptosis and autophagy, and play an integral role in a variety of diseases [13]. Several studies have shown the mTOR signalling pathway is closely related to the development of diabetes and concomitant liver injury [14,15].

**Figure 1:**
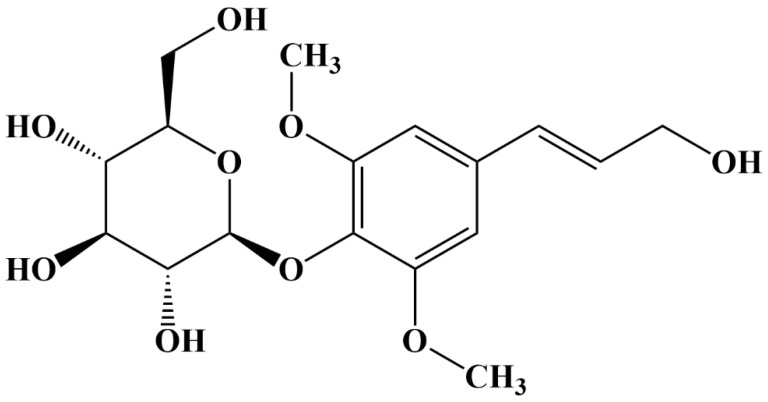
Molecular structure of EB.

Natural active products are rich in a variety of chemical compositions, which may have therapeutic effects through multiple targets and pathways compared to chemical drugs. This can effectively avoid the adverse effects triggered by a single target. In recent years, many natural products have been reported to have antidiabetic activity [16,17,18]. Acanthopanax senticosus is mainly found in the northeastern region of China. The roots, rhizomes, flowers, fruits, and leaves of this plant are used for medicinal purposes, with the effects of benefiting qi, strengthening the spleen, tonifying the kidney, and tranquilising the mind [19]. The discovery of the clinical efficacy of Acanthopanax senticosus and its wide application has recently led to a deeper understanding of its chemical composition, pharmacological effects, and clinical application. One of the main glycoside components of Acanthopanax senticosus is EB, a phenylpropanoid glycoside (molecular structure shown in Figure 1). Recent pharmacological studies have shown that EB has a wide range of pharmacological effects, including nephroprotective [20], anti-inflammatory [21], anti-tumour [22], and tranquilising [23] effects, and its safety and effectiveness have been demonstrated in long-term traditional applications. During the treatment phase, it was observed that EB at concentrations ranging from 50 to 150 μg∙mL^−1^ did not result in the death of any zebrafish. This indicates that EB is non-toxic within this concentration range.

Post-injection EB leads to a significant decrease in blood glucose levels in streptozotocin-induced diabetic Wistar rats, while also improving erythrocyte and leukocyte levels and their functional indices [24]. The combination of EB and tilianin was shown to exert anti-diabetic effects and to improve cardiac function and histopathological changes in diabetes-induced cardiomyopathy. Moreover, EB reduced the expression of NLRP3/IL-6/IL-1β/TNF-α and inhibited diabetes/hyperglycaemia-induced oxidative stress in rat heart and H9c2 cells [25]. However, systematic studies on the mechanism underlying the hypoglycaemic action of EB have yet to be reported.

In this study, we demonstrated that EB has hypoglycaemic, hypolipidaemic, and therapeutic effects on diabetic liver injury in a zebrafish model of diabetes. We used this model to search for possible markers of metabolic disorders and metabolic pathways through non-targeted metabolomic studies of the anti-diabetic effect of EB. This study provides an experimental basis for the clinical treatment of T2DM with EB and a theoretical basis for the development and utilisation of EB. This work may advance basic research into the treatment of DM.

## 2. Results

### 2.1. EB Improves Disordered Glucose and Lipid Metabolism in Zebrafish

**Figure 2:**
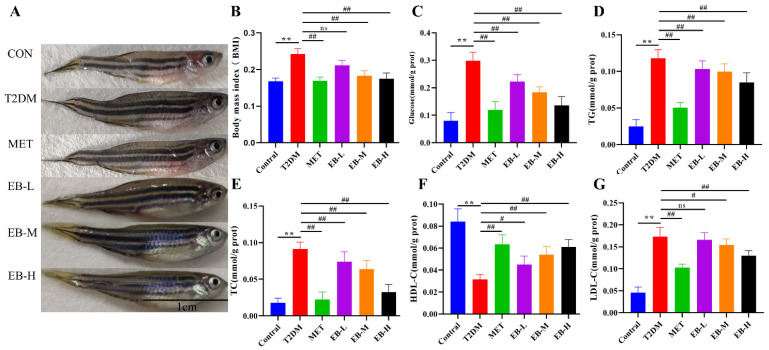
Whole-body biochemical indicators of six experimental groups of zebrafish. (**A**) Morphology of zebrafish before and after treatment. (**B**) BMI (*n* = 8). (**C**) Glucose content in zebrafish (*n* = 6). (**D**) Triglycerides (TG) (*n* = 6). (**E**) Total cholesterol (T-CHO) (*n* = 6). (**F**) High-density lipoprotein cholesterol (HDL-C) (*n* = 6). (**G**) Low-density lipoprotein (LDL-C) (*n* = 6). Data are expressed as the mean and SEM and were analysed by one-way analysis of variance (ANOVA), with Tukey multiple-comparison analysis. Compared with the control group, ** *p* < 0.01; compared with the model group, ^#^ *p* < 0.05, ^##^ *p* < 0.01 and ns for *p* > 0.05, not significantly different.

Obesity is a chronic inflammatory state due to excessive nutrient intake or metabolic disorders in the body and is a known cause of IR [26]. The prevalence of DM may be related to the degree and type of obesity, with centripetal obesity being the most relevant. The results of this study showed that zebrafish in the diabetes model group had a significantly higher BMI (*p* < 0.01) compared to the control group. The BMI of the Met, EB-M, and EB-H groups was significantly lower (*p* < 0.01) compared to the diabetes model group, but no significant difference was found with the EB-L group (*p* > 0.05) (Figure 2A,B). These results indicate that the obesity status of a zebrafish diabetes model improved after treatment with Met and with medium and high doses of EB.

Due to the small size of the 4-month-old zebrafish, blood sample collection was difficult to achieve. The head removal homogenisation method was therefore used to determine biochemical indices in the zebrafish. Since glucose metabolism and lipid metabolism are closely related, lipid metabolism disorders become more obvious with the development of diabetes. Clinical survey data show that about 40% of untreated diabetic patients suffer from hyperlipidaemia, of which 80% are diagnosed as hypertriglyceridemia [27]. The present study showed that combined feeding of glucose and cholesterol resulted in the development of hyperglycaemia and hyperlipidaemia features in the zebrafish diabetes model group, indicating the successful establishment of the model. Following treatment with the positive control drug metformin or with three different doses of EB, the zebrafish showed significantly reduced glucose content, TC, TG, and LDL-C (*p* < 0.01 or *p* < 0.05) and significantly increased HDL-C (*p* < 0.01 or *p* < 0.05) (Figure 2C–G). These results indicate that EB attenuates the abnormalities of glycolipid metabolism in zebrafish caused by high sugar and high fat. High-dose EB showed the best efficacy for improving the abnormalities of glycolipid metabolism.

### 2.2. EB Increases the Expression of the GLUT4 Protein and Alleviates Diabetes Complicated with Liver Injury

**Figure 3:**
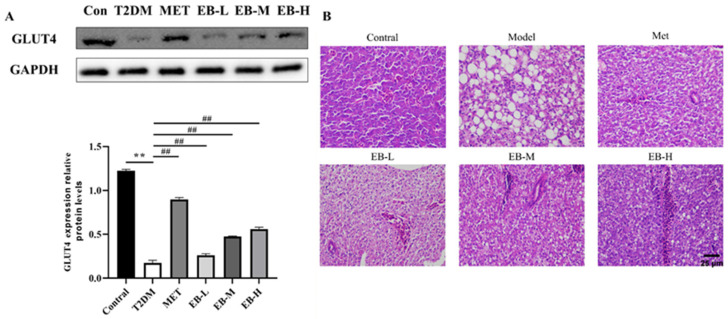
EB increases GLUT4 protein expression and reverses liver tissue damage in the zebrafish diabetes model. (**A**) Effect of EB on GLUT4 expression in zebrafish tissues. Protein expression levels were normalised to the levels of GAPDH. Data were analysed using a one-way ANOVA and expressed as the mean ± SEM. Compared with the control group, ** *p* < 0.01; compared with the model group, ^##^ *p* < 0.01. (**B**) H&E staining (scale bar, 25 μm; magnification, 400×).

Glucose transporter 4 (GLUT4) is a glucose transporter protein specifically expressed in skeletal muscle and adipose tissue. It is a key protein that affects glucose uptake by skeletal muscle cells and adipocytes and is closely linked to glucose metabolism [28]. GLUT4 protein expression was significantly lower (*p* < 0.01) in the diabetes model group compared to the control group (Figure 3). However, treatment with the positive control drug metformin and with all doses of EB significantly increased GLUT4 protein expression (*p* < 0.01) compared to the model group (Figure 3A).

DM is a metabolic disease characterised by hyperglycaemia. Prolonged exposure to hyperglycaemia causes glycation in the body and damage to various organs [29]. IR leads to enhanced hepatic gluconeogenesis and impaired hepatic lipid metabolism, resulting in liver injury [30]. As shown in Figure 3B, the liver tissue of zebrafish in the control group exhibited a dense structure, neatly arranged cells, abundant cytoplasm, and clear contours. In contrast, cells in the liver tissue of zebrafish from the model group were obviously atrophied, irregularly arranged, and had unclear boundaries. Moreover, large vacuoles and inflammatory cell infiltration were visible. Following treatment with different doses of EB, the vacuoles in the liver cells gradually reduced, the inflammatory cell infiltration decreased, and the arrangement of hepatocytes became more regular. The EB-H group in particular showed almost similar hepatocyte morphology to the control group (Figure 3B), suggesting that treatment with EB can effectively protect from liver injury caused by long-term hyperglycaemia.

### 2.3. Differential Metabolite Analysis

**Figure 4:**
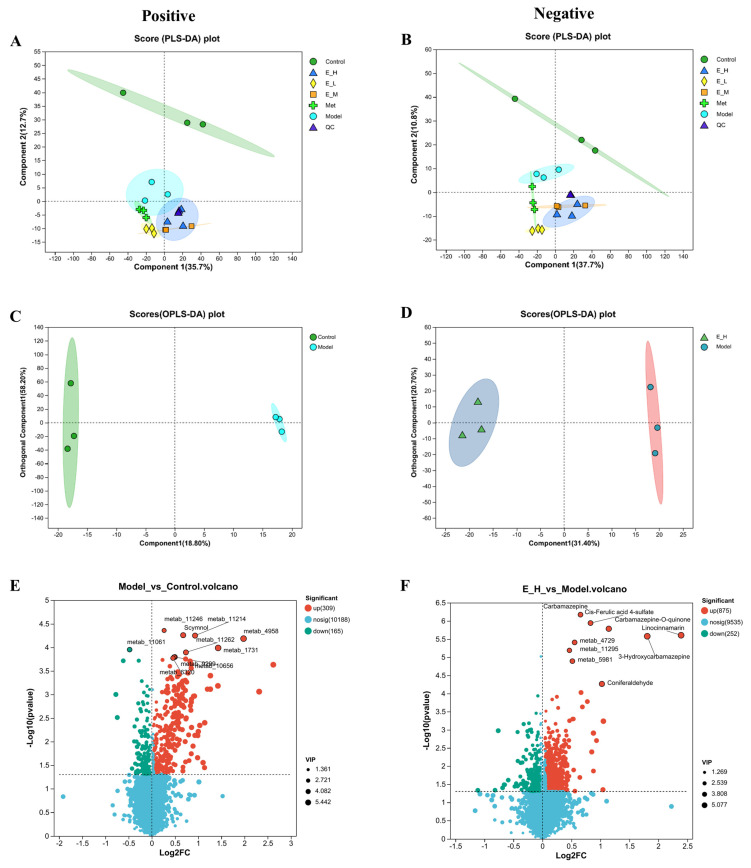
Multivariate statistical analysis. PLS−DA analyses of zebrafish samples from different treatment groups ((**A**): positive; (**B**): negative); OPLS-DA score plots ((**C**): control group vs. model group; (**D**): EB-H group vs. model group). Volcano plots, with points on the left side of the plots representing metabolites that were differentially down-regulated and points on the right side representing metabolites that were differentially up-regulated ((**E**): control vs. model group; (**F**): EB-H vs. model group).

A Partial Least Squares Discriminant Analysis (PLS-DA) model was employed to investigate differences in metabolite abundance between samples from each group. The PLS-DA score plots revealed the control group was completely separated from the experimental group, indicating the effects of high glucose and high fat could be observed at the metabolic level. Furthermore, separation of the two samples along the first principal component was significant, with cations and anions accounting for 35.7% and 37.7% of the total variance, respectively (Figure 4A,B). Orthogonal Partial Least Squares Discriminant Analysis (OPLS-DA) was also performed on the control group, the model group, and the EB-H group. This method reflects more intuitively the differences in the metabolic status of zebrafish. The OPLS-DA score plots revealed that the control and model groups were significantly differentiated, as well as the model and EB-H groups (Figure 4C,D). The samples within the group were relatively concentrated, indicating that EB had a regulatory effect on the in vivo metabolism of diabetic zebrafish.

The relative levels of metabolites in samples from different groups were compared and analysed. Metabolites that differed between groups were identified on the basis of VIP ≥ 1 and *p* < 0.05 and analysed by multiplicity of change differences. Differences in the content of metabolites between two groups were viewed by volcano plots, and the statistical significance was determined. The number of differential metabolites between the control and model groups was 95, comprising 56 up-regulated metabolites and 39 down-regulated metabolites. The number of differential metabolites between the model and EB-H groups was 273, comprising 167 up-regulated metabolites and 106 down-regulated metabolites (Figure 4E,F).

### 2.4. Metabolic Pathway Analysis

**Figure 5:**
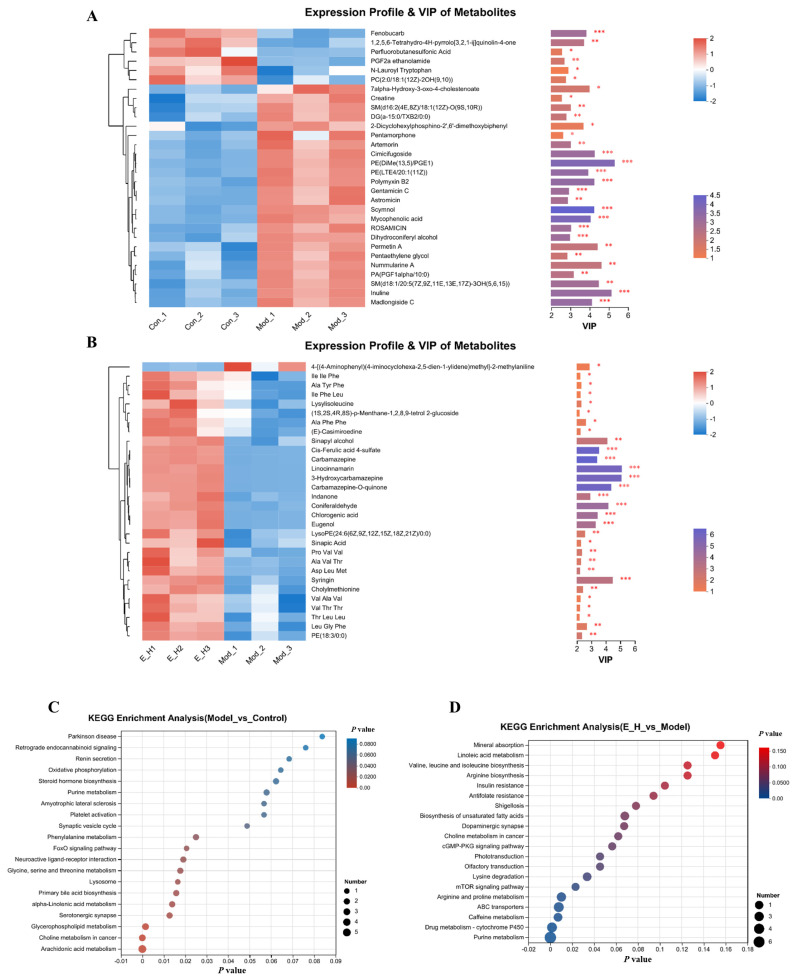
Heat map and metabolic pathway analysis. Heat map, * *p* < 0.05, ** *p* < 0.01, and *** *p* < 0.001 ((**A**): control group vs. model group; (**B**): EB-H group vs. model group). The horizontal coordinate is the enriched significance *p*-value, while the vertical coordinate is the KEGG pathway. ((**C**): control vs. model group; (**D**): EB-H vs. model group).

The VIP value reflects the importance of the variable in the classification. Differential variables with VIP values > 1 (considered statistically significant) were screened in the OPLS-DA model. Data for the screened differential variables were subjected to a one-way ANOVA *t*-test, and those with *p* < 0.05 were identified as being significantly different. This analysis showed that levels of Creatine, Artemorin, Cimicifugoside, Polymyxin B2, Gentamicin C, Astromicin, Scymnol, Mycophenolic Acid, Dihydroconiferyl Alcohol, Pentaethylene Glycol, and Nummularine A were significantly higher in the model group compared to the control group (Figure 5A).

In addition, the levels of Lysylisoleucine, L-Leucine, Carbamazepine, 3-Hydroxycarbamazepine, Carbamazepine-O-quinone, Prostavasin, Indanon, Coniferaldehyde, Chlorogenic Acid, Eugenol, Sinapic Acid, Cholylmethionine, and other substances were significantly higher in the EB-H group compared to the model group (Figure 5B). These results indicate the above endogenous substances may be potential markers for the pharmacological effects of EB in zebrafish.

KEGG enrichment analysis was performed for differential metabolites between the model and control groups, as well as between the EB-H and model groups. A total of 45 and 41 metabolic pathways were found to be enriched, respectively. The bubble diagrams for the top 20 ranked metabolic pathways are presented in Figure 5A. The differential metabolites between the control and model groups were mainly involved in arachidonic acid metabolism, glycerophospholipid metabolism, α-linolenic acid metabolism, the FoxO signalling pathway, the AMPK signalling pathway, and purine metabolism (Figure 5C). Differential metabolites between the model group and the EB-H treatment group were mainly involved in purine metabolism, cytochrome P450, caffeine metabolism, arginine and proline metabolism, the mTOR signalling pathway, insulin resistance, and glycerophospholipid metabolism (Figure 5D).

## 3. Discussion

Although the commonly used hypoglycaemic drugs for the treatment of DM have good effects, most also cause side effects in patients [31]. For example, rosiglitazone increases the risk of heart failure [32], while metformin causes adverse reactions such as diarrhoea [33] and nausea [34]. While it has been reported that EB lowers blood glucose [24], previous studies have focused mainly on the physiological changes induced by EB and less on the molecular mechanism of this hypoglycaemic effect.

Zebrafish was chosen in the present study as an experimental animal model in order to better understand the hypoglycaemic effect of EB and its mechanism of action. The major metabolism-related organs in zebrafish have a very similar structure to mammals, including the pancreas and some insulin-sensitive target tissues such as the liver and muscle. The similar regulation of glucose metabolism makes zebrafish a good model for diabetes research, and they are now widely used for the study of human metabolic diseases such as DM [35]. We used 1.75% glucose immersion combined with a high-fat diet to construct the zebrafish diabetes model. Although the induction period was long, this proved to be a stable model. The BMI and lipid indices in the model group were significantly different from those of the control group, indicating the intervention led to a disturbance of glucose and lipid metabolism and significantly increased the risk of metabolic disease.

Glycolipid metabolism plays an important role in the regulation of body lipids and is closely related to the development of T2DM [36]. Most patients with T2DM are obese and show disorders of glycolipid metabolism, such as elevated blood glucose, TG, and TC, with elevated LDL indexes but lower protective HDL indexes [37]. In the present study, the hypoglycaemic and lipid-lowering effects of EB were evaluated by comparing several physicochemical indices, such as BMI, glucose, TC, TG, LDL-C, and HDL-C, between different groups of zebrafish. The glucose content in diabetic zebrafish was significantly reduced in all EB treatment groups. Moreover, the EB treatment groups and the positive drug metformin showed good regulatory effects on blood lipid indexes in zebrafish.

The main physiopathological mechanism for the development of T2DM is related to IR. Effective improvement of IR is therefore critical to the treatment of diabetes [38]. GLUT4 is considered a key target for diabetes treatment since it is stimulated by insulin to transfer glucose from the outside of the cell and into muscle cells and adipocytes, thereby lowering the blood glucose level. Reduction of GLUT4 expression decreases the body’s sensitivity to insulin, thus causing IR [39]. Restoring the normal level of GLUT4 is therefore very important for enhancing glucose metabolism and improving IR. In this study, the combination of high glucose and a high-fat diet was found to significantly reduce GLUT4 expression in the zebrafish model group. Moreover, intervention with EB significantly increased the expression of GLUT4 and improved IR.

The prolonged hyperglycaemic state in diabetic patients causes damage and dysfunction in various tissues of the human body, especially the liver, eyes, kidneys, heart, and blood vessels, thus posing a serious risk to health [29]. In T2DM-induced liver injury, >90% of patients show non-alcoholic fatty liver disease, which is characterised by IR and hepatic fat accumulation. Recent studies have revealed that inflammation is generally implicated in liver diseases ranging from the initial to the late stages and is a common pathophysiological response to liver injury [40]. In the current study, we observed a reduction in hepatic glycogen vacuoles and hepatocyte damage after EB treatment, which may be related to its restoration of insulin signalling and attenuating the inflammatory response.

Using metabolomics, we found that EB caused significant changes in the levels of metabolites such as carbamazepine-O-quinone, DL-Acetylcarnitine, 5-Acetylamino-6-formylamino-3-methyluracil, and theobromine in zebrafish, all of which are closely related to glycolipid metabolism. In addition, intervention with EB increased the content of valine, leucine, and isoleucine. These branched-chain amino acids can promote fatty acid metabolism and reduce fat accumulation [41].

Balanced mTOR signalling activity is essential for coordinating nutrient and growth factor signalling with insulin sensitivity. Dysfunction of mTOR can cause T2DM and various complications [42], and impairment of the PI3K/Akt/mTOR pathway leads to IR [43]. The results of our metabolic pathway analysis indicate that the mechanism of action of EB for improving IR and regulating disordered glucose and lipid metabolism in diabetic zebrafish may be due to its regulation of the mTOR pathway.

Purines exist in the body mainly as purine nucleotides. These help to provide energy for metabolic processes, and their metabolites play a key role in regulating glucose and lipid metabolism. Disorders of lipid metabolism lead to abnormal adenine nucleotide metabolism, which then causes disorders of glucose metabolism through abnormal adenine nucleotide signalling. The results of our metabolomics analysis lead us to hypothesise that EB affects T2DM by regulating the level of purine metabolism.

There is growing evidence that caffeine is strongly associated with many chronic diseases, including T2DM, cardiovascular disease, and neurological disorders [44]. One study found that caffeine intake reduced the risk of cardiovascular disease in diabetic patients [45]. At the same time, prolonged caffeine intake was found to significantly decrease insulin secretion and increase beta-cell apoptosis, leading to potential abnormalities in glucose homeostasis [46]. Together with our metabolomic results, we hypothesise that EB may exert its hypoglycaemic effect by affecting caffeine metabolism.

Our metabolic pathway analysis also found that EB affects cytochrome P450, arginine and proline metabolism, IR, and the regulation of glycerophospholipid metabolism. This alleviates disordered glycolipid metabolism, thereby attenuating T2DM. Based on the findings of this work, further molecular biological studies are needed to confirm the detailed mechanism by which EB improves abnormal glucose and lipid regulation in energy metabolism. This may allow the rational application of EB and its preparations in the clinic.

In summary, this study systematically investigated the hypoglycaemic, hypolipidaemic, and hepatoprotective effects of EB in a diabetic zebrafish model. These activities may be related to purine, caffeine, arginine, and proline metabolism, as well as to the mTOR signalling pathway, IR, and glycerophospholipid metabolism. However, this paper still has limitations, such as the fact that the insulin levels of all groups of zebrafish were not measured and the mechanism of high glucose-induced liver damage was not investigated in depth, which will be added to future studies.

## 4. Materials and Methods

### 4.1. Drugs and Materials

EB (98%) was purchased from Chengdu Efa Biotechnology Co., Ltd. (Chengdu, China), glucose from Tianjin Tianli Chemical Co. (Tianjin, China), and cholesterol from Shanghai Yuanye Biotechnology Co. (Shanghai, China). Test kits for glucose, triglyceride (TG), total cholesterol (TC), LDL cholesterol (LDL-C), and HDL cholesterol (HDL-C) were purchased from Nanjing Jianjian Bioengineering Institute (Nanjing, China). Haematoxylin staining solution, eosin staining solution, and RIPA lysis solution were purchased from Shanghai Biyuntian Biotechnology Co. (Shanghai, China). Antibodies against GLUT4 and GAPDH were purchased from Cell Signalling Technology (Boston, MA, USA).

### 4.2. Animals and Experimental Design

Two-month-old zebrafish (AB-wild type) were purchased from Nanjing Yishu Pear Blossom Biotechnology Co. (Nanjing, China). This study was carried out in strict accordance with animal care guidelines stipulated by the Animal Experimentation Ethics Committee, Changchun University of Traditional Chinese Medicine. All efforts were made throughout the study to minimise animal suffering. The zebrafish culture water was selected from an E3 culture solution with a conductivity of 500 S/m. The water temperature was maintained at (28 ± 1) °C, with day and night cycles of 14 h of light and 10 h of darkness. The zebrafish were fed twice a day (8:00 a.m. and 5:00 p.m.), and for the first 7 days with plumpy shrimp to acclimatise. After they were able to swim freely and had acclimatised to the environment, they were fed with Zebrafish Diet No. 4. The zebrafish diabetes model was established as described previously by Gleeson [17], with slight modifications. To construct the zebrafish diabetes model, a 1.75% glucose immersion solution was combined with a high-fat diet containing 10% cholesterol.

**Figure 6:**
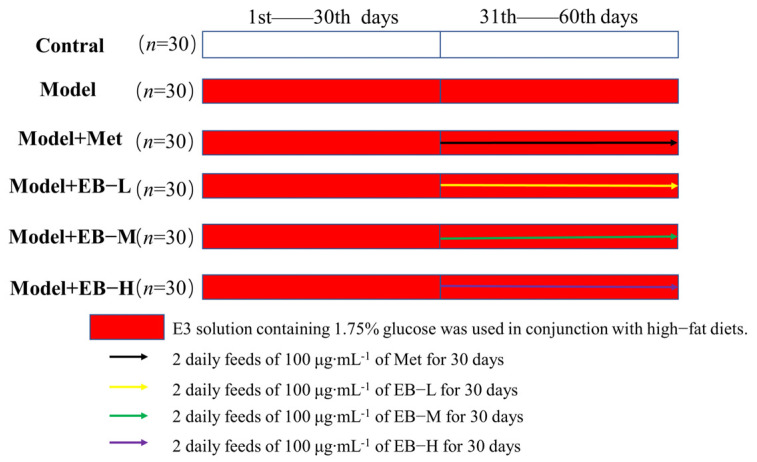
Experimental procedure.

A total of 180 zebrafish were randomly divided into control, model, metformin (100 μg∙mL^−1^), EB low (EB-L), EB medium (EB-M), and EB high (EB-H) doses (50, 100, and 150 μg∙mL^−1^, respectively) groups, with 30 fish in each group. The control group was grown with normal feed in E3 culture for 60 days. The model group was grown with high-fat feed in a 1.75% glucose E3 culture for 60 days. The metformin group and the EB-L, EB-M, and EB-H groups were grown with high-fat feed in a 1.75% glucose E3 culture for 60 days. The method for configuring the metformin group involved crushing and mixing metformin hydrochloride tablets, weighing out 100 mg of metformin, dissolving it in a litre of water, and stirring well. The resulting solution had a concentration of 100 μg∙mL^−1^. The corresponding therapeutic drugs were given by water-soluble exposure from the 31st day onward. The experimental flow is shown in Figure 6.

### 4.3. Sample Collection

Once they reached the treatment cycle, zebrafish were fasted for 12 h. When sampling, zebrafish were first removed and placed in trays filled with an ice–water mixture for freezing and sacrifice. The body surface was then quickly drained of water, and the fish was weighed and its length measured in a low-temperature environment. The remaining fish were snap-frozen in liquid nitrogen for preservation. The samples were then transferred from liquid nitrogen to −80 °C to be preserved for later examination.

### 4.4. Measurement of Biochemical Indexes

After anaesthetising the zebrafish, water was removed from the body surface using filter paper, and the fish were weighed using a 1/1000 scale. The distance from the tip of the head to the tip of the tail was measured accurately using a vernier calliper and recorded as the body length. Body mass index (BMI) was calculated using the formula: weight (kg)/body length squared (m^2^).

After the BMI was evaluated, the zebrafish were decapitated and homogenised. The tissues were accurately weighed, and a 9-fold volume of saline was added in the ratio of weight (g) to volume (mL) of 1:9. After mechanical homogenisation in a low-temperature environment, the tissue mixture was centrifuged at 4 °C and 2500 rpm for 10 min. The supernatant was removed for subsequent analysis. A biochemical kit was used to determine the protein concentration and levels of glucose, TC TG, HDL-C, and LDL-C in zebrafish tissues. The assay results were standardised according to the sample protein content.

### 4.5. Western Blot

Zebrafish tissues were sheared, and RIPA lysate containing 1% PMSF was added. They were then ground with a glass homogeniser, and the total protein was extracted by ultrasonication. Proteins were separated by SDS polyacrylamide gel electrophoresis and then transferred onto a PVDF membrane. This was blocked with 5% skimmed milk powder at room temperature for 1.5 h. Primary antibodies against GLUT4 and GAPDH were diluted (1:1000) with primary antibody diluent and incubated overnight at 4 °C on a shaker. The following day, the membrane was incubated with horseradish peroxidase-labelled goat anti-mouse/rabbit secondary antibody (1:5000) at room temperature for 1.5 h. ECL chemiluminescent solution was added dropwise to the membrane, and the proteins were visualised using a multifunctional gel imager. The grey scale value of protein bands was calculated with Image J software (version 1.48u). The grey scale value of the target protein band/GAPDH protein band indicated the relative protein expression level.

### 4.6. Histopathological Examination

Zebrafish liver tissues were collected, fixed with 4% paraformaldehyde, dehydrated with gradient ethanol, treated with xylene, and then dipped in wax. They were then embedded as pathological sections, stained with haematoxylin and eosin (HE), and sealed. Pathological changes in the liver tissues of each group were observed under the microscope.

### 4.7. Metabolite Extraction

A solid tissue sample (50 mg) was added to a centrifuge tube, and a diameter grinding bead was added. The sample was ground with a Wonbio-96c from Shanghai Wanbo Biotechnology Co., Ltd. (Shanghai, China) frozen tissue grinder for 6 min (−10 °C, 50 Hz) and then subjected to low-temperature ultrasonic extraction for 30 min (5 °C, 40 kHz) using 400 μL of extraction solution containing 0.02 mg/mL of internal standard (L-2-chlorophenylalanine). The samples were then left at −20 °C for 30 min, centrifuged for 15 min (4 °C, 13,000× *g*), and the supernatant transferred to the injection vial for LC-MS analysis. Preparation of a quality control sample to monitor analytical stability.

### 4.8. LC-MS Analysis

LC-MS/MS analysis was performed using a Thermo UHPLC-Q Exactive HF-X system equipped with an ACQUITY HSS T3 column (100 mm × 2.1 mm i.d., 1.8 μm; Waters, Milford, USA) from Majorbio Bio-Pharm Technology Co., Ltd. (Shanghai, China). The mobile phases consisted of 0.1% formic acid in water–acetonitrile (95:5, *v*/*v*; solvent A) and 0.1% formic acid in acetonitrile–isopropanol–water (47.5:47.5, *v*/*v*; solvent B). The flow rate was 0.40 mL/min, and the column temperature was maintained at 40 °C.

Mass spectrometric data were collected using a Thermo UHPLC-Q Exactive HF-X Mass Spectrometer. The optimal conditions were set as follows: an Aux gas flow rate of 13 arb, a sheath gas flow rate of 50 arb, a source temperature of 425 °C, and an ion-spray voltage floating at −3500 V in negative mode and 3500 V in positive mode. Normalised collision energy was set at 20−40−60 V rolling for MS/MS. The full MS resolution was 60,000, and the MS/MS resolution was 7500. Data acquisition was performed using Data Dependent Acquisition mode, with detection carried out over a mass range of 70–1050 *m*/*z*.

### 4.9. Data Anlaysis

The pretreatment of LC/MS raw data was performed by Progenesis QI software (version 3.0, Waters Corporation, Milford, CT, USA). A three-dimensional data matrix in CSV format was exported and included the metabolite name, sample information, and mass spectral response intensity. Internal standard peaks, as well as any known false positive peaks, were removed from the data matrix, deredundant, and peak pooled. Metabolites were identified by searching the Metlin (https://metlin.scripps.edu/, accessed on 28 December 2022), Majorbio, and HMDB (http://www.hmdb.ca/, accessed on 28 December 2022) databases.

The data matrix obtained from database searches was uploaded to the Majorbio cloud platform (https://cloud.majorbio.com, accessed on 28 December 2022) for analysis. It was first preprocessed as follows: at least 80% of metabolic features detected in each sample set were retained, and each metabolic signature was normalised. Moreover, variables of QC samples with an RSD > 30% were excluded and log10 logarithmicised in order to obtain the final data matrix for subsequent analysis.

Principal Component Analysis (PCA) and Orthogonal Partial Least Squares Discriminant Analysis (OPLS-DA) were then performed using ropls (version 1.6.2). Seven-cycle interactive validation was used to assess the stability of the model. Metabolites with VIP > 1, *p* < 0.05, were considered significantly different metabolites based on the variable importance in projection (VIP) values obtained from the OPLS-DA model and the *p*-value generated by the Student’s *t*-test.

Differential metabolites between two groups were mapped to their corresponding biochemical pathways using metabolic enrichment and pathway analysis based on the KEGG database (http://www.genome.jp/kegg/, accessed on 28 December 2022). Enrichment analysis was performed using the Python (version 1.0.0) package ‘scipy.stats’ (https://docs.scipy.org/doc/scipy/, accessed on 28 December 2022) to identify the most relevant biological pathways for experimental treatments.

### 4.10. Statistical Analysis

SPSS (version 21.0) was used for statistical analysis, with results obtained from the data represented as mean ± SEM. Comparisons between multiple groups were made using one-way ANOVA, and differences were considered statistically significant when the *p*-value was ≤0.05.

## 5. Conclusions

EB has a hypoglycaemic effect and significantly improves disorders of glucose and lipid metabolism in a diabetic zebrafish model, as well as improving diabetes-induced liver injury. The mechanism of action may involve regulation of the mTOR signalling pathway, purine metabolism, caffeine metabolism, and glycerophospholipid metabolism. These findings indicate that EB is a new preventative and therapeutic agent for T2DM. However, further in-depth research and validation of the specific links in the metabolic pathways involved are still required. This study provides an experimental basis for the clinical treatment of T2DM with EB and a theoretical basis for the development and utilisation of EB.

## Data Availability

Data will be made available on request.

## Abbreviations

EB: eleutheroside B; DM, diabetes mellitus; T2DM, type 2 diabetes mellitus; TG, triglyceride; TC, total cholesterol; HDL-C, HDL cholesterol; LDL-C, LDL cholesterol; BMI, body mass index; and GLUT4, glucose transporter 4.
